# Modulation of Gamma-Secretase for the Treatment of Alzheimer's Disease

**DOI:** 10.1155/2012/210756

**Published:** 2012-12-19

**Authors:** Barbara Tate, Timothy D. McKee, Robyn M. B. Loureiro, Jo Ann Dumin, Weiming Xia, Kevin Pojasek, Wesley F. Austin, Nathan O. Fuller, Jed L. Hubbs, Ruichao Shen, Jeff Jonker, Jeff Ives, Brian S. Bronk

**Affiliations:** Satori Pharmaceuticals, Inc., 281 Albany Street, Cambridge, MA 02139, USA

## Abstract

The Amyloid Hypothesis states that the cascade of events associated with Alzheimer's disease (AD)—formation of amyloid plaques, neurofibrillary tangles, synaptic loss, neurodegeneration, and cognitive decline—are triggered by A**β** peptide dysregulation (Kakuda et al., 2006, Sato et al., 2003, Qi-Takahara et al., 2005). Since **γ**-secretase is critical for A**β** production, many in the biopharmaceutical community focused on **γ**-secretase as a target for therapeutic approaches for Alzheimer's disease. However, pharmacological approaches to control **γ**-secretase activity are challenging because the enzyme has multiple, physiologically critical protein substrates. To lower amyloidogenic A**β** peptides without affecting other **γ**-secretase substrates, the epsilon (**ε**) cleavage that is essential for the activity of many substrates must be preserved. Small molecule modulators of **γ**-secretase activity have been discovered that spare the **ε** cleavage of APP and other substrates while decreasing the production of A**β**_42_. Multiple chemical classes of **γ**-secretase modulators have been identified which differ in the pattern of A**β** peptides produced. Ideally, modulators will allow the **ε** cleavage of all substrates while shifting APP cleavage from A**β**_42_ and other highly amyloidogenic A**β** peptides to shorter and less neurotoxic forms of the peptides without altering the total A**β** pool. Here, we compare chemically distinct modulators for effects on APP processing and *in vivo* activity.

## 1. Introduction

Gamma-secretase (*γ*-secretase) is required for the production of amyloid beta peptides (A*β*) and decreasing A*β* production as a disease modifying approach for the treatment of Alzheimer's disease (AD) has received intense interest. The initial focus was on the discovery of compounds that would decrease *γ*-secretase activity. *γ*-Secretase cleaves the membrane bound C-terminal domain (C99) of APP at the *ε* site to produce the intracellular domain, AICD. The enzyme then makes sequential cuts of the remaining intramembrane APP fragment at each turn of the alpha helix (every 3-4 amino acids) until A*β* peptides are formed and released into the extracellular space [1–3]. This protein processivity produces A*β* peptides that vary in size, from 43–34 amino acids in length [4, 5]. In Alzheimer's disease, a greater number of the longer forms of A*β*, including A*β*_42_ and A*β*_43_, or a high ratio of the long peptides to the shorter forms, appear to occur [6]. These longer A*β* peptides readily oligomerize, forming toxic species, as well as becoming the seeds for amyloid plaques [7, 8].

The full inhibition of *γ*-secretase appeared to be a sound approach. However, it was found that *γ*-secretase plays a broader biological role and cleaves multiple proteins to yield physiologically essential products. Thus, total inhibition results in severe adverse effects *in vivo *[9–11]. This played out in the clinic in the trial of the *γ*-secretase inhibitor, semagacestat from Eli Lilly [12–14]. Patients treated with this drug developed skin and gastrointestinal side effects that are characteristic of the inhibition of *γ*-secretase processing of Notch, leading to the discontinuation of the clinical trial in 2010 [13, 14].

The discovery of compounds that could decrease the production of the more amyloidogenic A*β*_42_ peptide while preserving total A*β* levels and *γ*-secretase cleavage of other substrates led to a clinical trial of one of these newly identified, first generation gamma-secretase modulators (GSMs) [15]. The NSAID-derived, Flurizan from Myriad Genetics was tested in a Phase 3 trial in mild to moderate AD patients. However, Flurizan is a very weak modulator of *γ*-secretase, with an IC_50_ of ~250 *μ*M [16]. In addition, this compound has very poor distribution into the central nervous system [16, 17]. Not surprisingly, the combination of these less than desirable properties resulted in no exposure in the brain, and a failed Phase III trial in June 2008 [18].

Second and now third generation GSMs have been discovered and are proceeding toward clinical trials in AD. These GSMs are significantly more potent than Flurizan and appear to have better drug-like properties. However, the majority of these compounds fall into one of two chemical classes, with little structural diversity within each of these classes and the development of some has been discontinued because of toxicities and biopharmaceutical limitations that other class members may also share [19]. A structurally unique GSM derived from a core molecule isolated from a natural product is also moving through preclinical testing [20]. While all three chemical series of GSMs share some common pharmacological properties, they differ in other fundamental ways. Here we present data contrasting the pharmacology of members of several structural series of GSMs and how modulation may avoid the pitfalls associated with *γ*-secretase inhibitors (GSIs).

## 2. Materials and Methods

### 2.1. Test Compounds

LY411575, GSI-953, BMS-708163, MK-GSM1, JNJ-40418677, and E-2012 were prepared according to published methods. SPI-1802 and SPI-1810 were prepared at Satori Pharmaceuticals.

### 2.2. Cell Culture and Compound Treatment

SUP-T1 cells (ATCC) were cultured in T75 flasks in RPMI media (Mediatech 10-041-CV) supplemented with 10% FBS and penicillin/streptomycin at 37°C in a 5% CO_2_ atmosphere. One hour prior to drug treatment, six well plates were seeded with 1.5 mL of media containing 2% FBS and cells at a density of 1.5 × 10^6^ cells/mL. Test compounds in DMSO were diluted 100-fold directly into the media with the cells and incubated for 18 hours at 37°C. After treatment 100 *μ*L aliquots of treated cells were assayed for viability with the Promega Cell Titer Glo assay system. The conditioned media and cells were further processed to measure A*β* levels and NICD levels, respectively.

CHO-2B7 cells (Mayo Clinic) are Chinese hamster ovary cells stably transfected with human *β*APP 695 wt [38, 39]. The cells were cultured in Ham's F12 media (Thermo Fisher SH30026.01) supplemented with 10% FBS, 0.25 mg/mL Zeocin and penicillin/streptomycin at 37°C in a 5% CO_2_ atmosphere. For compound treatment, cells were plated in 96-well plates at a density of 1.0 × 10^5^ cells/mL and allowed to grow to 100% confluence over two days. Test compounds in DMSO were diluted 100-fold directly into the media before adding to the cells. Immediately prior to adding compound-containing media to the cells, they were washed once with 1XPBS. Conditioned media from CHO-2B7 cells were collected after 5 hours of treatment and the levels of A*β* peptides were assessed as described below.

H4 human neuroglioma cells (ATCC) were cultured in 10% FBS/DMEM (Media Tech) with Pen/Strep (50 units/50 *μ*g/mL; Invitrogen). Human WT APP stably transfected CHO cells were cultured in 10% FBS/HAM'S F-12 growth media (Media Tech) supplemented with Pen/Strep and G418 (500 *μ*g/mL; Promega). Cells were plated and grown to confluency in 96-well plates prior to dosing. Cells were washed with PBS and 100 *μ*L of media containing DMSO alone (vehicle) or test compounds in DMSO at a final DMSO concentration of 1% (v/v). Conditioned media was collected after 18 hours of treatment and diluted 1 : 1 with MSD blocking buffer (1% BSA in MSD wash buffer).

### 2.3. Solid Phase Extraction

Wells of 30 mg Oasis HLB 96-well extraction plates (Waters Corporation) were activated by addition of 1 mL of methanol followed by rinsing with 1 mL of water utilizing a vacuum plate manifold. 1 mL of SUP-T1 conditioned media was added and wells were then washed sequentially with 2 mL of 10% methanol and then with 2 mL of 30% methanol. Samples were eluted into sample collection tubes by adding 250 *μ*L of 90% methanol with 2% ammonium hydroxide to each well. Eluted samples were concentrated to dryness under vacuum without heating.

### 2.4. A*β In Vitro* Assay Measurement

Conditioned media was collected after 5–18 hours of treatment and diluted with 1 volume of MSD blocking buffer (1% BSA in MSD wash buffer). Alternatively, dried films of SUP-T1 conditioned media after solid phase extraction were resuspended with 1 volume of MSD blocking buffer (1% BSA in MSD wash buffer). Samples were transferred to blocked MSD Human (6E10) A*β* 3-Plex plates and incubated for 2 hours at room temperature with orbital shaking followed by washing and reading according to the manufacturer's instructions (SECTOR Imager 2400 Meso Scale Discovery, Gaithersburg MD).

### 2.5. NICD Assay

The remaining cells were washed twice in PBS and then lysed with Promega reporter lysis buffer containing a complete protease inhibitor cocktail (Roche) for 1 hour at 4°C. Lysates were spun at 5,000 RPM for 5 minutes and supernatants were collected. Total protein levels were measured and adjusted to 1-2 mg/mL total protein using the BCA total protein assay (Thermo Scientific). NICD levels were then measured with a cleavage specific Notch1 sandwich ELISA (Cell Signaling Technologies) according to the manufacturer's instructions.

### 2.6. Immunoprecipitation and Matrix-Assisted Laser Desorption/Ionization Time-of-Flight (MALDI-TOF) Mass Spectrometry

Chinese Hamster Ovary cells stably transfected with wild-type human APP were treated for 6 hrs with *γ*-secretase modulators at an approximate concentration of 10-fold the IC_50_ (Merck GSM1 at 1 *μ*M, JNJ-40418677 at 2 *μ*M, SPI-1802 at 3 *μ*M, and SPI-1810 at 2 *μ*M.) Monoclonal A*β* antibodies 6E10 (specific for amino acids 1–16 of A*β*) and 4G8 (specific for amino acids 17–24 of A*β*; Covance, Dedham, MA) were immobilized with agarose resin using the AminoLink Plus reagents (Thermo Scientific, Rockport, IL). Conditioned media from treated cells was precleared with agarose resin overnight, and the supernatant was incubated with agarose-conjugated 6E10/4G8 for 6 hrs. Immunoprecipitates were washed extensively prior to analysis.

Analyses were performed on a Shimadzu Biotech Axima TOF2 (Shimadzu Instruments) matrix-assisted-laser desorption/ionization time-of-flight (MALDI-TOF) mass spectrometer. Peptides were analyzed in positive ion linear mode. For intact peptide mass measurement the instrument was set with a mass range extending up to 6000 m/z using a pulsed extraction setting of 3500. An average mass external standard was used which consisted of angiotensin II (1047.2), P14R (1534.86) and ACTH clip 18–39 (2465.20), insulin B (3496.67), and insulin (5734.51). For sample preparation, 5 *μ*L aliquots of A*β* containing immunoprecipitates were diluted with 10 *μ*L of 0.1% TFA and then desalted using a C18 Zip Tip (Millipore, Corp.). Samples were directly deposited from the Zip Tip onto the MALDI sample target and then mixed with 0.5 *μ*L of matrix solution which consisted of 5 mg/mL of alpha cyano-4-hydroxy cinnamic acid in acetonitrile: 0.1% TFA (50 : 50). Data was acquired manually using a set laser power and averaging 1500–2000 laser shots. 

### 2.7. *In Vivo* Study Methods

All animal handling and procedures were conducted in full compliance to AAALAC International and NIH regulations and guidelines regarding animal care and welfare. 

Either transgenic mice (Tg2576, 3 mos; *N* = 21) or wild-type Sprague Dawley rats (200–225 g; *N* = 8) were utilized to assess *in vivo* efficacy. All animals were acclimated to the test facility for a minimum of two days prior to initiation of the study. Compounds were dosed orally in 10 : 20 : 70 Ethanol/Solutol/Water via oral gavage. Samples were harvested at 6 hrs after dose for A*β* and compound exposure levels. Blood samples were collected into K2EDTA and stored on wet ice until processed to plasma by centrifugation (3500 rpm at 5°C) within 30 minutes of collection. Each brain was dissected into three parts: left and right hemispheres and cerebellum. Brain tissues were rinsed with ice cold phosphate buffered saline (without Mg^2+^ or Ca^2+^), blotted dry and weighed. Plasma and cerebella were analyzed for parent drug via LC/MS/MS. Parent drug levels were compared to a standard curve to establish the unknown levels. 

### 2.8. Rodent A*β* Determination

This protocol is a modification of protocols described by Lanz et al. [40] and Rogers et al. [41]. Frozen hemispheres were weighed into tared homogenization tubes (MP Biomedicals#6933050 for rat; MP Biomedicals, Solon, OH) and (Simport#T501-4AT; Simport, Beloeil, Qc, Canada) containing one 5 mm stainless steel bead (Qiagen#69989) for mouse). For every gram of brain, 10 mLs of 6 M guanidine hydrochloride (wild-type rat) or 0.2% diethyl amine in 50 mM NaCl (transgenic mouse) was added to the brain-containing tubes on wet ice. Rat hemispheres were homogenized for one minute and mouse hemispheres were homogenized for 30 seconds at the 6.5 setting using the FastPrep-24 Tissue and Cell homogenizer (MP Biomedicals#116004500). Homogenates were rocked for two hours at 4°C, then precleared by ultracentrifugation at 100,000 ×g for one hour at 4°C. Precleared wild-type rat homogenates were concentrated over solid phase extraction (SPE) columns (Oasis HLB 96-well SPE plate 30 um, Waters#WAT058951; Waters Corp., Milford, MA). Briefly, SPE columns were prepared by wetting with 1 mL of 100% methanol followed by dH_2_O using vacuum to pull liquids through. Brain homogenates were then added to the prepared columns (1.0 mL from rat). Columns were washed twice with 10% methanol followed by two washes with 30% methanol. Labeled eluent collection tubes (Costar cluster tubes #4413; Corning Inc., Corning, NY) were placed under SPE columns and samples were eluted under very mild vacuum with 300 *μ*L of 2% NH_4_OH/90% methanol. Eluents were dried to films under vacuum with no heat in a speed vacuum microcentrifuge. Films were resuspended in 150 *μ*L of Meso Scale Discovery (MSD, Gaithersburg MD) blocking buffer (1% BSA in MSD wash buffer) for one hour at room temperature with occasional vortexing. A volume of 45 *μ*L of precleared transgenic mouse brain homogenates were diluted into 450 *μ*L of blocking buffer and were neutralized with 5 *μ*L of 0.5 M Tris pH 6.8. For A*β*38, 40, and 42 measurements, MSD 96 well multispot Human/Rodent (4G8) A*β* triplex ultrasensitive ELISA plates were blocked with MSD blocking buffer for 1 hour at room temperature with orbital shaking. A volume of 25 *μ*L of neat resuspended wild-type rat brain homogenate films or diluted transgenic mouse brain homogenates were added in duplicates to the blocked 3-plex A*β* MSD plates with SULFO-TAG 4G8 antibody (MSD). The A*β* 3-Plex plates were incubated for 2 hours at room temperature with orbital shaking followed by washing and reading according to the manufacturer's instructions (SECTOR Imager 2400, MSD). The average A*β* concentrations from duplicate measurements of each animal were converted to percent vehicle values and the treatment group averages were statistically compared by ANOVA analysis. 

## 3. Results and Discussion

**Figure 1:**
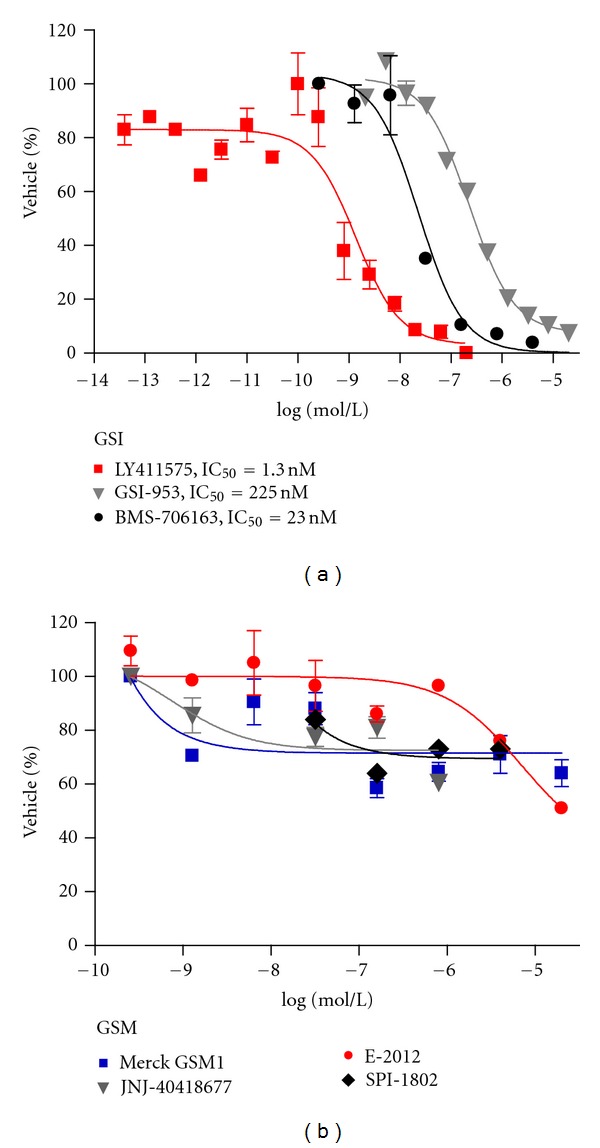
Multiple GSI and GSMs were examined in for their ability to inhibit NOTCH cleavage using the SUP-T1 cellular assay.

*γ*-Secretase is a complex enzyme with multiple substrates and multiple cleavage sites on at least some of these substrates, including APP. Complete inhibition of *γ*-secretase activity by targeting the *ε* cleavage site prevents the processing of multiple physiologically relevant proteins, leading to the severe side effects reported in AD patients [12, 13]. On the other hand, chemically modulating the enzyme with a GSM is a more precise mechanism to enhance certain cleavage events while preventing the cut that yields the amyloidogenic peptide, A*β*_42_ which is linked to the pathophysiological initiation of AD (Figure 6). In preclinical toxicological testing, GSMs appear to be free of the mechanism-based toxicities attributed to the inhibition of Notch processing that have plagued the enzyme inhibitors. *In vitro* data demonstrate that Notch cleavage to NICD is not inhibited by any of several represented GSMs at concentrations that do not disrupt cell viability (Figure 1). Treatment with GSMs in rodents have not shown the classical Notch-related toxicities that are associated with GSIs, suggesting that the complete processing of Notch to NICD can occur in the presence of GSMs. 

### 3.1. GSMs Do Not Show a Potency Shift with Changes in Substrate Concentration

*γ*-Secretase inhibitors can show a significant shift in potency in APP transfected cell-based assays depending on the level of expression of APP.  Table 1 compares the IC_50_ for A*β*_42_ lowering of different GSIs in multiple cell lines with varying levels of APP expression. In the highest expressing cell lines, the GSIs assayed here appear more potent (lower IC_50_s in the high expressing, transfected CHO-2B7 cell line) than when the same compounds are tested in assays using lower expressing cell lines (native H4 cell line). This potency shift seen with GSIs has been attributed to a shift in the enzyme/substrate ratio [42], where there is a higher ratio in wild-type cells versus those that overexpress APP. This higher ratio in turn requires more compounds to inhibit the enzymatic reaction. Interestingly, the potency shift due to substrate concentration that occurs with GSIs does not occur with GSMs. As seen in Table 1, the IC_50_ for all the GSMs shown are consistent across cell lines, regardless of the APP expression levels. While this potency shift is not well understood, these data do suggest that GSIs and GSMs affect the enzyme in fundamentally different ways.

### 3.2. Structural Diversity of GSMs

**Figure 2:**
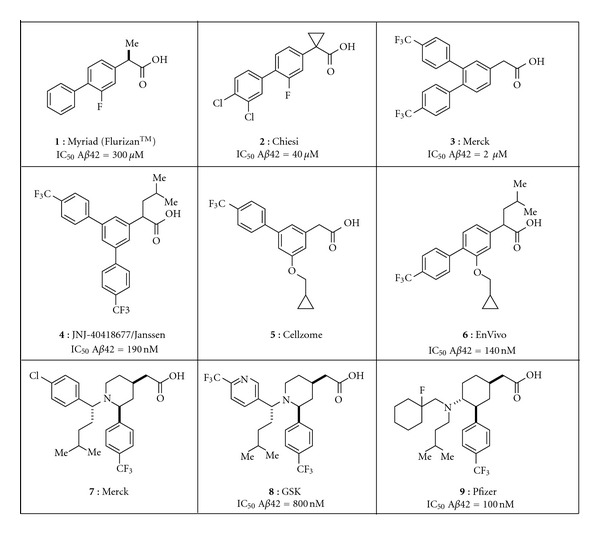
Representative NSAID-inspired GSMs. Compounds **1–6** are aryl acetic acids [21–26], compounds **7** and **8** are piperidine acetic acids [27, 28] and compound **9** is a cyclohexane acetic acid [29].

Many of the second generation GSMs were inspired by the NSAIDs (Figure 2). For instance, all compounds shown in the figure were initially derived from the aryl acetic acid motif found in **1** and similar NSAIDs [21]. The similarity between these compounds makes for a crowded patent landscape, with some compounds potentially covered in multiple patent applications from different sponsors. In addition, for this structural series, the physicochemical properties indicate that all of these compounds carry an increased safety risk due to the high logP, low PSA [43–45], and high degree of aromaticity [46, 47]. Compounds **7–9** are designed to improve upon these properties, but are still considered high risk due to these same physicochemical based *in silico* models [27–29]. *In vivo* preclinical toxicity testing will ultimately be needed to assess the safety profile of these similar structures. However, the lack of progression of compounds from this class into and through clinical development suggests that this scaffold may have challenges that will continue to slow or prevent successful conduct of the studies required for registration.

**Figure 3:**
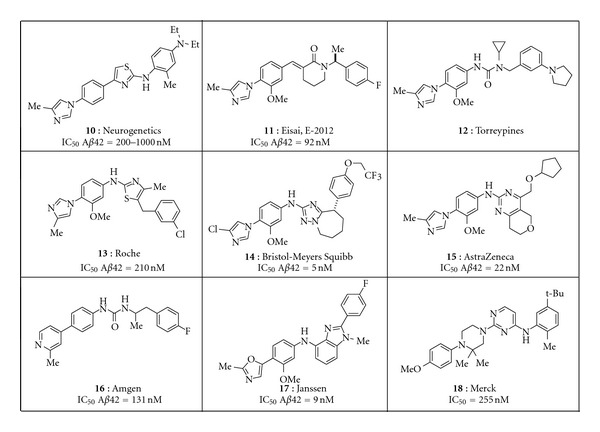
Representative aryl imidazole inspired GSMs [30–33]. Compounds **16–18** are the most unique because the aryl imidazole has been replaced by a bioisostere [34–36].

A second class of GSMs that has received significant attention is summarized in Figure 3. Following initial disclosure of **10** and **11**, a number of pharmaceutical companies pursued structurally related chemical series [30, 48]. Although this class offers clear distinction from the NSAID-inspired compounds above, their physicochemical properties also reside outside of the molecular space most frequently affiliated with marketed agents for oral therapy. For example, the number of aromatic rings and clogP of the representatives shown are higher than the average for oral compounds on the market [46, 47], leading to a higher probability of safety and biopharmaceutical challenges with this class. Although some groups have been successful in developing promising structural alternatives, as exemplified by **16–18**, little has been reported on the development of any representatives from this general scaffold [34–36].

**Figure 4:**
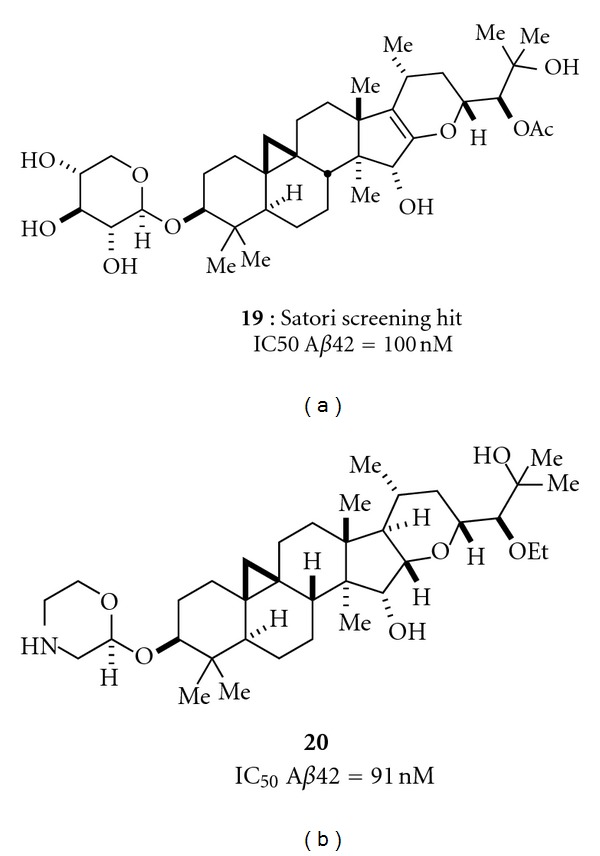
Two examples of Satori GSMs.

A novel and structurally distinct chemical architecture of a third class of GSMs has been reported by Satori Pharmaceuticals. This scaffold was first isolated from black cohosh, leading to the characterization of initial hit **19** (Figure 4) [20]. A combination of synthetic and medicinal chemistry optimization led to **20**, which is reported to have better drug properties than **19** [20]. To date, the compounds reported by Satori also fall outside of the guidelines most typically associated with good *in vivo* disposition. The group notes, however, that the majority of marketed agents derived from natural products also violate these same guidelines, a trend that has led some to conclude that molecules derived via semisynthesis on natural product scaffolds define a different set of guidelines. Compounds from this class are now proceeding through preclinical development.

### 3.3. A*β* Peptide Profiling of Structurally Diverse GSMs

**Figure 5:**
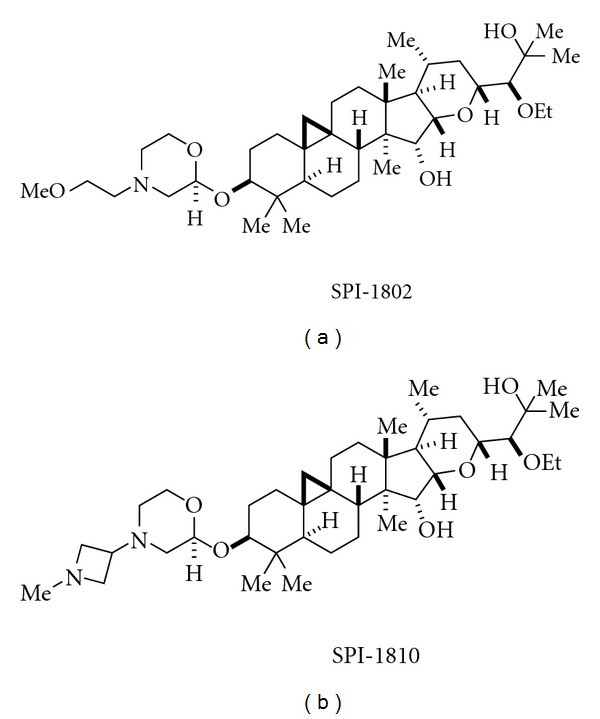
Structures of SPI-1802 and SPI-1810 [20, 37].

**Figure 6:**
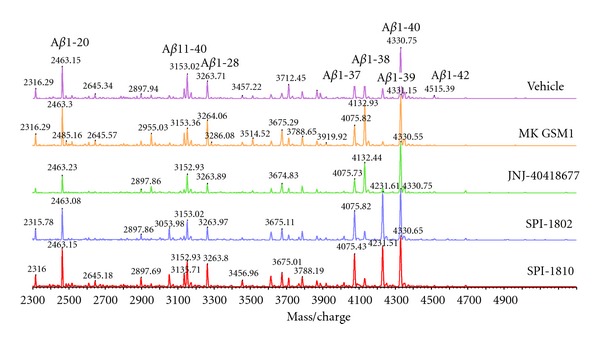
MALDI-TOF analysis of A*β* peptides from conditioned media of APP-overexpressing CHO cells treated with GSMs. The immunoprecipitated A*β* peptides were subjected to MALDI-TOF analysis to visualize individual A*β* fragments. Using a combination of two A*β* antibodies, 6E10, and 4G8 allows precipitation of full length and N- and C-terminal truncated A*β* peptides.

When the NSAID-type of GSMs was first described (Figure 2), the changes in A*β* peptides that were seen, a decrease in A*β*_42_, an increase in A*β*_38_ and little or no change on A*β*_40_ or total A*β* were labeled the “modulator profile,” nomenclature that was reinforced by the pharmacology reported for the GSMs represented by the structures in Figure 3. However, since that time, additional A*β* peptide profiles have been reported for the chemically distinct scaffold disclosed by Satori Pharmaceuticals, as well as molecules more closely related to those in Figure 3. Examples of the variety of A*β* peptide profiles produced by GSMs are shown in Figure 6. Merck GSM1 and JNJ-40418677 lower A*β*_42_ while increasing A*β*_38_, with little or no effect on A*β*_40_. Alternatively, the Satori Pharmaceutical compounds, SPI-1802 and SPI-1810 (structures shown in Figure 5) decrease both A*β*_42_ and A*β*_38_, but maintain total A*β* levels by increasing A*β*_37_ and A*β*_39_. Yet, all of these compounds can be classified as “gamma secretase modulators” based on the commonality of sparing the *ε* cleavage of C99 and other substrates (e.g., Notch), decreasing A*β*_42_, and not affecting total A*β* levels. 

Based on these data, modulation of *γ*-secretase is more accurately defined as a shift of the A*β* pool to shorter, but variable length, A*β* peptides. A physiological role for A*β* peptides has not been discovered, but *in vitro *studies have demonstrated that shorter peptides are incapable of aggregation and oligomerization [19] and may even prevent the oligomerization of A*β*_42_ by binding to it, suggesting that preserving the total pool of A*β* may be beneficial. 

The molecular mechanism by which GSMs modulate *γ*-secretase activity is not completely understood. The variety of A*β* peptide profiles that result from treatment of cells with different *γ*-secretase modulators suggests that the enzyme has numerous allosteric modulatory sites. These are not necessarily completely unique sites and published data suggests the sites may be overlapping [49]. The modulator binding sites thus far identified all seem to reside on the presenilin component of *γ*-secretase [50]. 

### 3.4. *In Vivo* Activity of GSMs

*In vivo*, GSMs exhibit the same A*β* profile as observed *in vitro* with reductions in A*β*_42_ and increases or decreases of other A*β* peptides and no effect on total A*β* levels. However, the amount of compound required to generate these effects has been surprisingly high when compared to the *in vitro* potency of the molecules. For example, the Eisai GSM E-2012 has an *in vitro* IC_50_ of 33 nM in a cell-based assay. *In vivo*, 4.9 *μ*M plasma concentrations of the compound were required to reduce brain A*β* by 25%. Similarly, Merck GSM1, JNJ-40418677, and SPI-1810 also require high plasma levels in order to achieve 25% lowering (Table 2), a factor that does not appear to be readily attributable to plasma protein binding. These high compound levels are not required for *in vivo* efficacy with GSIs which further suggests that the interaction with *γ*-secretase by a GSM is different from that of a GSI. Why high concentrations of some GSMs are required to modulate *γ*-secretase activity *in vivo* is not completely understood. Less than ideal pharmacokinetic properties, such as low free fraction or poor blood brain barrier permeability may contribute to the need for high plasma concentrations of GSMs to see efficacy in the brain *in vivo*. In addition, there are data indicating that GSMs can bind to both active and inactive forms of presenilin since the binding site of the modulators is available even prior to endoproteolysis that creates the active form of *γ*-secretase [51, 52]. Conversely, data indicate that GSIs require complex formation prior to binding [53], which may mean more binding sites are available for GSMs than for GSIs. 

## 4. Conclusions

**Figure 7:**
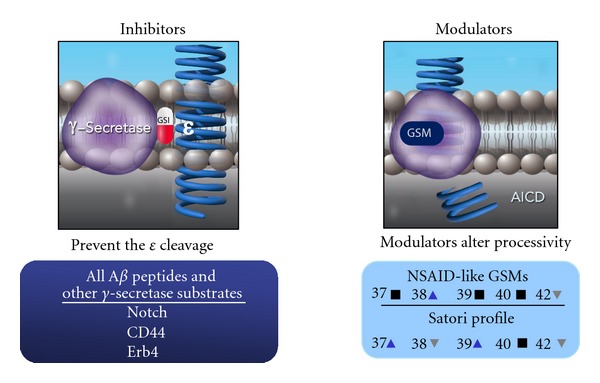
Diagram highlighting the differences between inhibitors and modulators.

Small molecule modulators of *γ*-secretase are now in the early stages of clinical testing. In preclinical toxicology studies, these modulators are free of the mechanism-based toxicities that have been seen with GSIs, most of which appear to be due to inhibition of Notch processing by directly targeting the *ε* cleavage site. Both *in vitro* and *in vivo* data support the conclusion that GSMs do not interfere with Notch processing, but instead, via slight shifts in the cleavage site on APP, lower A*β*_42_ production to produce the other normal A*β* peptide products (Figures 6 and 7). Thus, modulation of *γ*-secretase may represent the most selective approach to treating Alzheimer's disease via a decrease in production of A*β*_42_, more selective that an inhibitor of either beta or *γ*-secretase. Modulators do differ both in chemical structure and in their effects on APP processing, producing the desired decrease in A*β*_42_ and a variety of changes in other A*β* peptides. Understanding the molecular basis of these varying profiles may shed further light on the biology of *γ*-secretase. 

## Figures and Tables

**Table 1:** GSMs do not show a potency shift with APP overexpression.

Cell line	H4	H4-APP	CHO-SW	CHO-7W	CHO-2B7
A*β*_42_ Levels (pg/mL)	20	110	131	834	200

Inhibitors	A*β*_42_ IC_50_ (nM) per cell line				

LY411575	1.40	1.20	0.20	ND	0.05
GSI-953	706	52	5.20	8.70	2.50
BMS-708163	40	8	0.98	1.10	0.20

Modulators	A*β*_42_ IC_50_ (nM) per cell line				

GSM1	54	64	154	73	62
JNJ-40418677	115	133	190	122	172
E-2012	42	84	54	36	33

**Table 2:** GSM potency versus plasma exposure in mice.

	E-2012	JNJ-40418677	GSM1	SPI-1810
*In vitro* A*β*_42_ IC_50_ (nM) CHO-2B7 cells	33	172	62	114
Plasma exposure (*μ*M)	4923	7764	2744	14638
Plasma fold over A*β*_42_ IC_50_ for 25% reduction	149	45	44	128
Brain exposure (*μ*M)	2749	7497	8681	20368
Brain fold over A*β*_42_ IC_50_ for 25% reduction	83	44	140	179
